# Mass mortality event in South American sea lions (*Otaria flavescens*) correlated to highly pathogenic avian influenza (HPAI) H5N1 outbreak in Chile

**DOI:** 10.1080/01652176.2023.2265173

**Published:** 2023-10-19

**Authors:** Mauricio Ulloa, Antonio Fernández, Naomi Ariyama, Ana Colom-Rivero, Carlos Rivera, Paula Nuñez, Paola Sanhueza, Magdalena Johow, Hugo Araya, Juan Carlos Torres, Paola Gomez, Gabriela Muñoz, Belén Agüero, Raúl Alegría, Rafael Medina, Victor Neira, Eva Sierra

**Affiliations:** a Veterinary Histology and Pathology, Institute of Animal Health and Food Safety, Veterinary School, University of Las Palmas de Gran Canaria, Las Palmas de Gran Canaria, Spain; b Servicio Nacional de Pesca y Acuicultura, Valparaíso, Chile; c Programa de Doctorado en Ciencias Silvoagropecuarias y Veterinarias, Universidad de Chile, Santiago, Chile; d Departamento de Medicina Preventiva Animal, Facultad de Ciencias Veterinarias y Pecuarias, Universidad de Chile, Santiago, Chile; e Servicio Agrícola y Ganadero, Santiago, Chile; f Escuela Medicina Veterinaria, Facultad de Recursos Naturales y Medicina Veterinaria, Universidad Santo Tomas, Santiago, Chile; g School of Medicine, Pontificia Universidad Católica de Chile, Santiago, Chile; h Department of Pathology and Laboratory Medicine, School of Medicine, Emory University, Atlanta, GA, USA

**Keywords:** Mortality, South American sea lions, strandings, highly pathogenic avian influenza H5N1, outbreak, Chile

## Abstract

In Chile, since January 2023, a sudden and pronounced increase in strandings and mortality has been observed among South American (SA) sea lions (Otaria flavescens), prompting significant concern. Simultaneously, an outbreak of highly pathogenic avian influenza H5N1 (HPAIV H5N1) in avian species has emerged since December 2022. To investigate the cause of this unexpected mortality, we conducted a comprehensive epidemiological and pathologic study. One hundred sixty-nine SA sea lions were sampled to ascertain their HPAIV H5N1 status, and long-term stranding trends from 2009 to 2023 were analyzed. In addition, two animals were necropsied. Remarkably, a significant surge in SA sea lion strandings was observed initiating in January 2023 and peaking in June 2023, with a count of 4,545 stranded and deceased animals. Notably, this surge in mortality correlates geographically with HPAIV outbreaks affecting wild birds. Among 168 sampled SA sea lions, 34 (20%) tested positive for Influenza A virus, and 21 confirmed for HPAIV H5N1 2.3.4.4b clade in tracheal/rectal swab pools. Clinical and pathological evaluations of the two necropsied stranded sea lions revealed prevalent neurological and respiratory signs, including disorientation, tremors, ataxia, and paralysis, as well as acute dyspnea, tachypnea, profuse nasal secretion, and abdominal breathing. The lesions identified in necropsied animals aligned with observed clinical signs. Detection of the virus *via* immunohistochemistry (IHC) and real-time PCR in the brain and lungs affirmed the findings. The findings provide evidence between the mass mortality occurrences in SA sea lions and HPAIV, strongly indicating a causal relationship. Further studies are needed to better understand the pathogenesis and transmission.

## Introduction

Since its identification in China in 1996, highly pathogenic avian influenza (HPAI) caused by H5N1, has spread throughout the world causing an epizootic that has affected Africa, Asia and the Pacific, the Americas, Europe, and the Middle East. The virus has continually threatened not only wild, captive, and free-range birds, but also wild, captive, and domestic mammals, as well as humans (Kandeil et al. 2023).

In Chile, the HPAIV H5N1 emerged in December 2022 (Ariyama et al. 2023) and further, an alarming rate of mortality and morbidity in wild birds and marine mammals has been observed. Chile has 16 administrative regions of which 15 are coastal. To date, the virus has been detected in the 16 administrative Regions of the country. On February 15th, 2023, the first case of HPAI H5N1 2.3.4.4b clade was confirmed in *Otaria flavescens,* commonly named as South American (SA) Sea Lion, in the Antofagasta Region. With an estimated national population of 125,000 individuals, and its presence spanning the entire territory (Oliva et al. 2020; Venegas et al. 2002), *Otaria flavescens,* poses a significant potential risk of virus dissemination throughout all regions of the country.

To date, some reports described HPAI in marine mammals, especially in pinnipeds (seals and sea lions). The reported species included harbor seals (*Phoca vitulina*); gray seals (*Halichoerus grypus*) (Mirolo et al. 2023; Puryear et al. 2023) in the northern hemisphere and more recently, in *Otaria flavescens* in Peru (Gamarra-Toledo et al. 2023; Leguia et al. 2023). Gamarra-Toledo et al. reported a mass mortality of 3,108 SA sea lions on Peruvian coasts and observed neurological clinical signs that the authors hypothesized that these events could be related to acute HPAIV H5N1 infections. The HPAI H5 2.3.4.4b clade in pinnipeds and related to mass mortality events increases concern for animal and public health. The ability of HPAI H5N1 to evolve generating mammalian adaptation increases the concern of the human population. In addition, deceased infected pinnipeds can act as a source of the virus to other species. Therefore, we performed a long-term analysis of strandings of SA sea lions from 2009 up to June 2023 and conducted an epidemiological and pathologic investigation to elucidate the cause of this sudden mortality including testing for HPAIV H5N1.

## Materials and methods

### Data collection

The historical data regarding stranding events of SA sea lions in Chile were acquired from the data set of the National Fisheries and Aquaculture Service (SERNAPESCA) of Chile. The information encompasses the period from January 2009 to June 2023 and comprises georeferenced points, precise locations, the number of SA sea lions affected in each event, and, when available, additional data such as size, sex, age, body condition, visible lesions, and possible causes of stranding. For the analysis, we considered mortality and strandings as the same, due to strandings in more than 95% of the cases finalized in death. Regarding determining the moment when the stranding of pinnipeds arises statistically significant, statistical process control (SPC) charts were performed using the number of deceased animals per month, considering the whole country (Thor et al. 2007; Ilieş et al. 2020).

### HPAI molecular detection

From January 2023, samples from deceased sea lions were collected by veterinarians of the SERNAPESCA. The standard sampling includes a tracheal swab, using a 45 cm swab, but on occasions was complemented with rectal swabs and fresh feces. Each animal was tested individually but the tracheal and rectal swabs were pooled. The samples were collected in denaturant viral transport media (KaiBiLi™ Extended NTM, The Hague, Netherlands), refrigerated at 4 °C, and submitted for real-time RT-PCR at the SAG Livestock Virology Laboratory and Biotechnology Laboratory (at SAG, Lo Aguirre).

The samples were processed within the next two days after arrival. The RNA was obtained using the MagMAX™ Core Viral/Pathogen kit (Thermofisher, AM1830), and the first identification was attempted using the VetMAX-Gold AIV Detection Kit (Applied Biosystems™ Cat No. 4485261) for generic Influenza A virus (IAV) detection, targeting the M gene. From positive animals, real-time RT-PCR and Sanger sequencing were attempted for H5 2.3.4.4b clade confirmation using VSL-USDA protocols (NVSL 2023a, 2023b, 2023c).

### Spatial analysis

Georeferenced locations of live strandings, carcasses, and confirmed positive cases of *O. flavescens* were used for high-risk spatial cluster determination. In addition, publicly available data regarding positivity to HPAIV in avian sources (non-poultry species) was also used, determining high-risk spatial clusters to evaluate geographical relationships (https://www.sag.gob.cl/ia). The presence of spatial clusters or ‘hotspots’ of cases was identified utilizing Scan Statistics, specifically Kulldorff’s space exploration statistic, considering a purely spatial analysis with a Bernoulli distribution (Rao et al. 2017). Point and cluster maps were then generated using the QGIS (QGIS Association 2023) and SatScan software (Kulldorff 2022), allowing for visual representation and a better understanding of the spatial patterns associated with the HPAIV cases. Statistical significance of the spatial clusters was considered for p-values < 0.05.

### Clinical observations and necropsy

Two specimens of SA sea lions were necropsied, and tissue samples were collected for molecular diagnosis, histopathology, and immunohistochemistry. The animals were found in the coastal area of Iquique City, Tarapaca Region. Both specimens were found stranded and not able to recover, and after clinical observation, they were euthanized using a combination of Ketamine –Xylazine for sedation followed by the injection of T61 solution. The necropsy was conducted *in situ* by personnel from SERNAPESCA and SAG. Gross lesions were carefully documented, and samples of fresh and formalin-fixed tissues were collected, including the brain, pancreas, liver, lungs, urinary bladder, and kidneys. Furthermore, nasal, tracheal, and rectal swabs were also collected using the same protocol previously described for HPAI molecular detection. The necropsied animals were afterward buried to avoid the virus spreading.

### Immunohistochemistry

Selected fixed tissues including the brain, lungs, heart, and pancreas were processed for Hematoxylin and Eosin (HE) staining and Immunohistochemistry (IHC) targeting the Influenza Nucleoprotein. These analyses were performed at the Institute for Animal Health and Food Safety of The University of Las Palmas de Gran Canaria. Briefly, for IHC, 3-µm-thick FFPE tissue sections that were deparaffinized, hydrated, and subjected to antigen retrieval (the sections were treated with proteinase K for 6 min). Endogenous peroxidase activity was blocked (EnVision FLEX Mini Kit, High pH, Dako) for 10 min. A mouse primary monoclonal antibody (EBS-I-238; Biologicals Limited, https://biologicals-ltd.comExternal Link) targeting the nucleoprotein of all strains of influenza virus type A was used at a dilution of 1.5 µg/ml for 30 min at room temperature (RT). The slides were then incubated with an indirect peroxidase polymeric detection kit (EnVision FLEX Mini Kit, High pH, Dako) for 30 min at RT, followed by revelation with Magenta solution (HRP Magenta, Dako). Slides were counterstained with hematoxylin, coverslipped, and examined microscopically. The immunostaining included a positive reference control (brain sample molecularly confirmed positive for H5N1) and a negative control (the same section in which a primary antibody was omitted).

In addition, molecular techniques were also performed from some formalin-fixed paraffin-embedded (FFPE) samples. More in detail, RNA was extracted from four 10 μm-thick FFPE sections of the cerebrum of the male and the cerebellum of the female SA sea lions and deparaffinized using the xylene-alcohol method according to the manufacturer’s recommendations (RNeasy FFPE Kit, Qiagen, Inc., Valencia, CA, USA) with some modifications. Specifically, the washing steps involved in xylene deparaffinization were performed twice at 50 °C for 3 min, followed by a twice washing with ethanol after residual xylene was removed and overnight digestion at 37 °C. Molecular detection of type A influenza virus (a real-time reverse transcriptase PCR (RRT-PCR) assay based on the avian influenza virus matrix gene with a set of primers and a TaqMan probe) and the avian H5 and H7 Hemagglutinin subtypes (with two probe sets developed based on North American avian influenza virus sequences) was performed as previously described (Spackman et al. 2002). Additionally, molecular detection of morbilliviral nucleic acid was performed by a 1-step reverse transcription PCR of a 426-bp conserved region of the phosphoprotein gene with universal morbillivirus primers, as described (Barrett et al. 1993).

## Results

### Mass mortality on South American sea lions in Chile

Stranding events were divided into two categories, single and multiple stranding events, single events comprise only one individual while multiple events comprise more than one individual at a time. From 2009 to 2022, an average of 205 [SD = 98.6] annual stranding events were reported, involving 313 [SD = 177] stranded individuals of SA sea lions per year. Surprisingly, from January 2023 until June 2023, a total of 4,861 stranding events comprising 14,705 individuals were reported, representing an increase of 1,983% per year (in half a year). From 2009 to 2019, the mortality rate among stranded South African sea lions was 27%. However, in 2023, 95% of stranded sea lions were reported as deceased, these differences were statistically significant.

**Figure 1.**
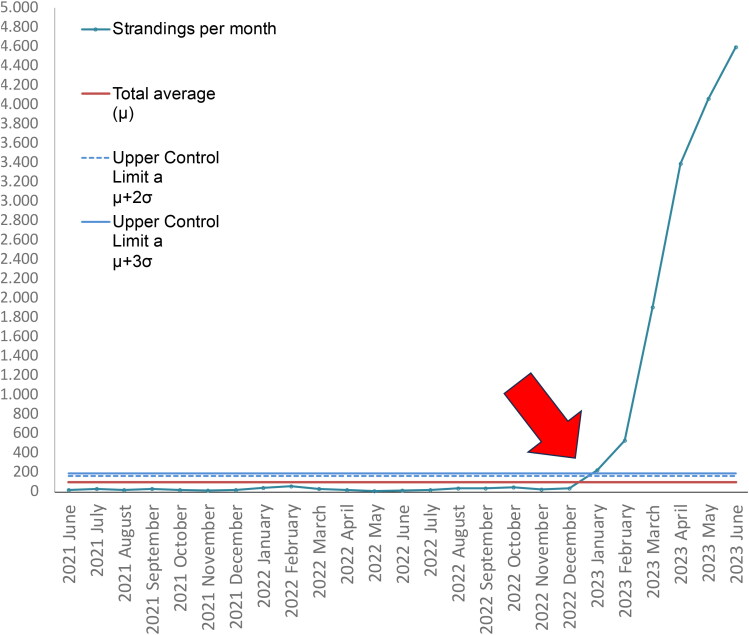
Statistical process control chart of *Otaria flavescens* strandings by month was collected from January 2009 to June 2023 (Illustrated from January 2020). the red arrow depicts January 2023 where the stranded surpasses the upper control limits.

**Figure 2.**
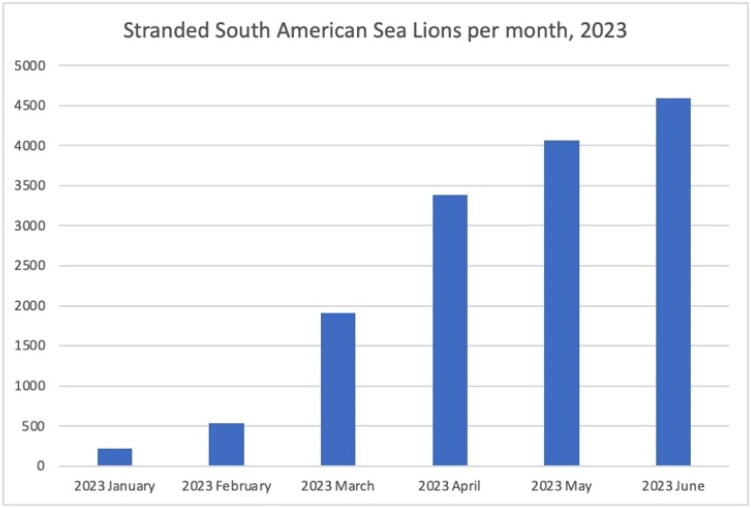
Column graph showcasing the frequency of stranded *Otaria flavescens* (South American sea lion) throughout the year 2023 by month.

The SPC chart shows the increase in the stranding of sea lions in January 2023, surpassing the upper control lines of two and three standard deviations over the average (Figure 1). In January 2023, a total of 223 stranded SA sea lions were documented. Subsequently, over the following months, a notable logarithmic increase was observed. This trend reached a peak in June 2023, with 4,594 stranded animals being registered (Figure 2). An increase of stranded animals was observed in all 15 coastal Chilean regions, but it was focused on the Northern Chilean regions that includes Arica, Tarapaca, Antofagasta, Atacama, and Coquimbo regions with a range of 669 to 4,626 between January and June 2023. In addition, the Bio-Bio region in Southern Chile, 600 km south of the Northern regions also has evidence of a high number of stranded animals (*n* = 507). The summary of results can be retrieved from Table 1.

**Table 1.** Table of South American sea lion Strands until June 30, 2023, Chile.

Region	January	February	March	April	May	June	Total
Arica	1	167	690	918	919	704	3,399
Tarapacá	1	6	238	423	555	489	1,712
Antofagasta	4	28	275	859	1,450	2,010	4,626
Atacama		7	155	412	629	302	1,505
Coquimbo	5	42	134	141	150	197	669
Valparaiso	7	6	20	35	4	1	73
O’Higgins			60	150	12		222
Maule	1		30	82	24	10	147
Ñuble		6	13	23	6	7	55
Bio-Bio	2	3	141	193	139	29	507
Araucania		1	3	10	6		20
Los Rios	1		4	7	7	1	20
Los Lagos	1	3	1	20	24	5	54
Aysen					1	4	5
Magallanes		1	1	2	3	4	11
Total General	23	270	1,765	3,275	3,929	3,763	13,025

### Detection of HPAI H5N1 in individuals

From January 8^th^ to June 30^th^, 152 stranding cases of SA sea lions, involving 168 SA sea lion individuals were sampled for Influenza A detection by real-time RT-PCR. The complete list is presented in Supplementary Table S1. So far, out of 168 animals, 34 have been confirmed positive for Influenza A virus by real-time RT-PCR, which represent 20% of the animal sampled. The Ct values from swabs ranged between 20 to 35, with a mean of 30.1 [SD = 4.4]. The samples collected from positive specimens were either oropharyngeal or rectal samples, most of them processed in pools. To date, positive HPIAV H5N1 confirmed cases have been detected in 12 out of the 15 coastal Regions of Chile. Twenty-one of the 34 IAV-positive cases were identified and confirmed as H5N1 2.3.4.4b clade, the remaining cases were inconclusive due to concerns of sample quality of high Ct values in the IAV detection.

### Distribution of high-risk spatial clusters

**Figure 3.**
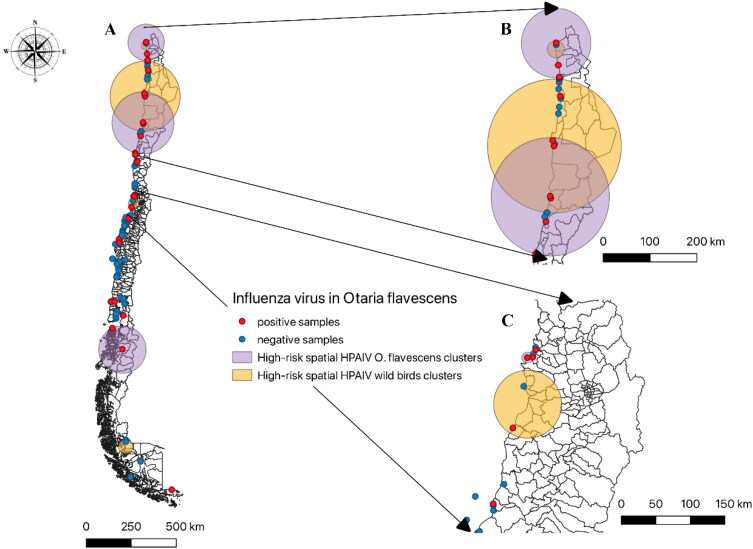
Spatial distribution and the high-risk cluster of influenza virus (IV) positive and negative samples from *O. flavescens* in Chile. **A** National situation of HPAIV, showing positive and negative samples (red and blue dots respectively) and high-risk spatial clusters for *O. flavescens* (light purple circles), and high-risk spatial clusters from wild bird outbreak in Chile (light brown circle). **B** overlaying high-risk significant HPAIV from wild birds and *O. flavescens* located in northern Chile. **C** proximity between statistically significant high-risk HPAIV spatial clusters for wild birds and positive cases of HPAIV in *O. flavescens* in coastal areas of central Chile.

There is a noticeable correlation between the widespread mortality of SA sea lions and the occurrences of HPAIV in wild birds. This correlation is particularly evident when analyzing the geographic distribution of cases. Figure 3 displays a significant overlap of high-risk spatial clusters, indicating a connection between cases of SA sea lions and wild birds, primarily in northern Chile. Additionally, the presence or absence of SA sea lions further accentuates this correlation, with statistically significant high-risk spatial clusters among wild birds.

A remarkable finding emerges from the analysis of SA sea lions. An observed positivity for IAV in SA sea lions is statistically significant (*p* = 0.0081) and showcases a high-risk spatial cluster with a relative risk of 3.96. This cluster is situated at coordinates 18.419067 S − 70.321289 W and spans a radius of 202.72 km.

### Pathological findings in necropsied specimens

We performed a necropsy on two specimens. The first one corresponded to a subadult male with good body condition with an estimated body weight of 70–80 kg. In the clinical observation, we observed neurologic clinical signs including tremors, stumbling, stiffness of the neck, nystagmus, weakness, and paralysis of limbs. In addition, dyspnea with predominantly abdominal breathing was observed (Video 1). The main gross lesions observed in this animal were highly hyperemic lungs with atelectasis and emphysematous areas, and in the brain, there was a marked hyperemic appearance in the vessels of the groves suggestive of meningoencephalitis inflammation. In this case, the virus was identified by real-time RT-PCR in several tissues including the brain (Ct: 18.4), lungs (Ct: 26.8), liver (Ct:30.6), kidney(Ct: 27.3), urinary bladder (Ct: 25.2), and heart (Ct: 31.4). The lowest Ct value was observed in the brain (Ct = 18.4). However, this animal was negative in oral, nasal, and rectal swabs.

**Figure 4.**
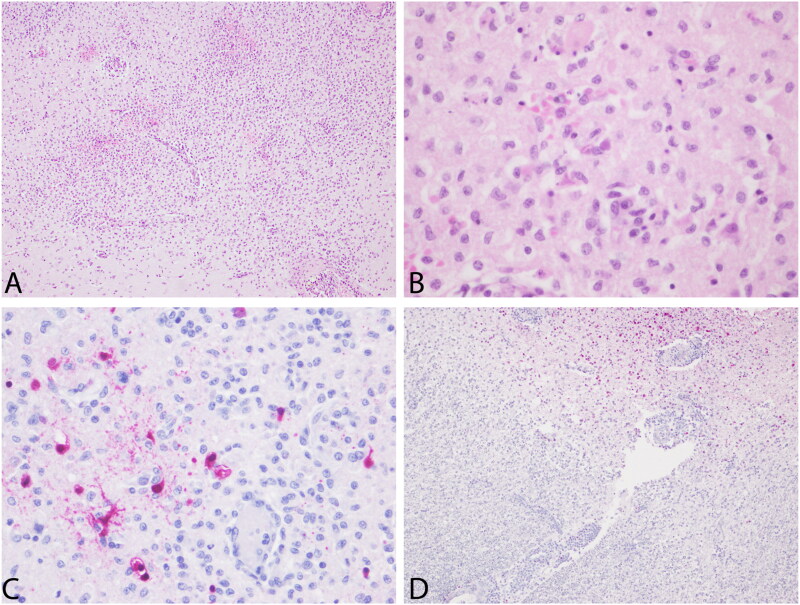
A. Brain. Male South American (SA) sea lion (245513). severe multifocal to locally extensive areas of non-suppurative encephalitis and hemorrhages. H&E, 10×. B. Brain. Male South American (SA) sea lion (245513). neuronal and glial necrosis with associated neuronophagia and gliosis. H&E, 40×. C. Brain. Male SA sea lion (245513). Intralesional positive immunostaining. IHC against Influenza A nucleoprotein in neurons and glial cells, IHC against Influenza A nucleoprotein, 40×. D. Arbor vitae of the cerebellum. Female sea lion (245719). Intralesional positive immunostaining. IHC against Influenza A nucleoprotein, 10×.

**Figure 5.**
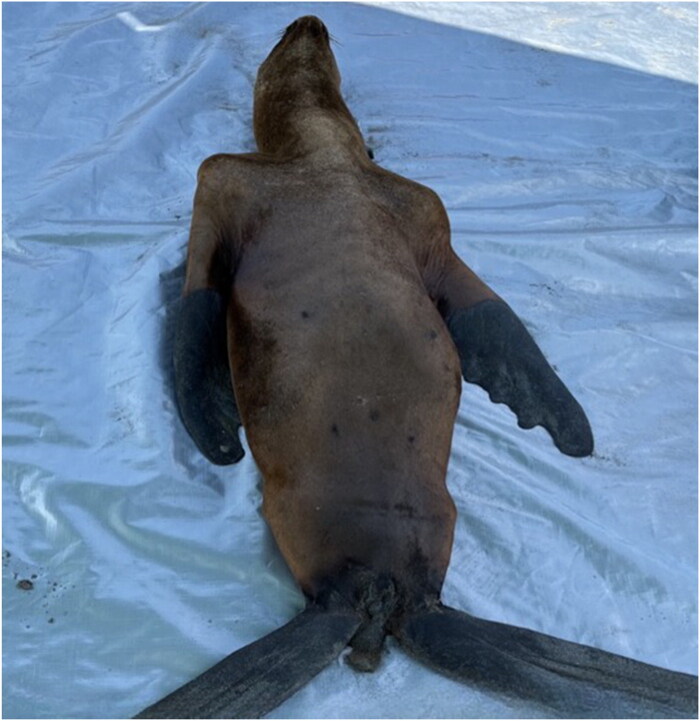
The *Otaria flavescens* (South American sea lion) adult female necropsied. The animal tested positive for the HPAI H5N1 virus. The SA sea lion displayed a prominent wasting syndrome, characterized by inadequate body condition and muscular weakness, with notably pronounced ribs reflecting its poor physical state.

Microscopically, focal isolated pneumonia and bronchial dilation with a protein-rich edematous exudate and fresh blood as well as few peri-bronchial multifocal foci of cell debris, epithelial-like cells and inflammatory cells involving the glandular area were observed in the lungs. No other lesions were histologically detected in the pancreas and heart tissue sections. However, severe multifocal to locally extensive non-suppurative meningoencephalitis and encephalitis, mostly affecting the gray matter, with a large number of lymphohistiocytic and plasmacytic perivascular cuffs, endothelial cells activation, and abundant gliosis, neuronophagia, and neuronal and glial necrosis (characterized by fragmentation and acidophilia) associated with hemorrhages were observed (Figure 4A and B). The other animal corresponded to an adult female with poor body condition. In the clinical observation, we have evidence of severe weight loss, muscle wasting, emaciation, and weakness (Figure 5). No evident neurologic clinical signs were observed in this animal (Video 2). The main gross lesions observed in this animal were congestion of the liver and mesentery vessels together with enlarged mesenteric lymph nodes. Contrary to the male, the female presented the virus in rectal (Ct: 32.7) and tracheal swabs (Ct: 31.9), lungs (Ct: 21), pancreas (Ct: 31.9), liver (Ct: 23.8), urinary bladder (Ct:33.7), and heart (Ct:34.8), but negative to the brain. The histopathological study showed emphysema and exudate in the bronchial lumen of lung tissue sections as well as marked multifocal emphysema with mucous exudate involving neutrophilic and eosinophilic cells within the bronchial lumen (including nematodes in one bronchus). In the heart, focal necrotizing myocarditis, without clear inflammatory cell infiltration, was observed. Similarly, a focal extensive necrotizing pancreatitis, affecting the exocrine portion, was found. Regarding the brain, moderate focal lymphohistiocytic and plasmacytic focal mononuclear meningitis and encephalitis characterized by perivascular cuffing, gliosis, and neuronal necrosis, were the main findings in the cerebrum. In addition, extensive gliosis, perivascular cuffing, neuronal degeneration, and necrosis with the presence of cellular debris and associated hemorrhages were detected in the arbor vitae of the cerebellum.

**Figure 6.**
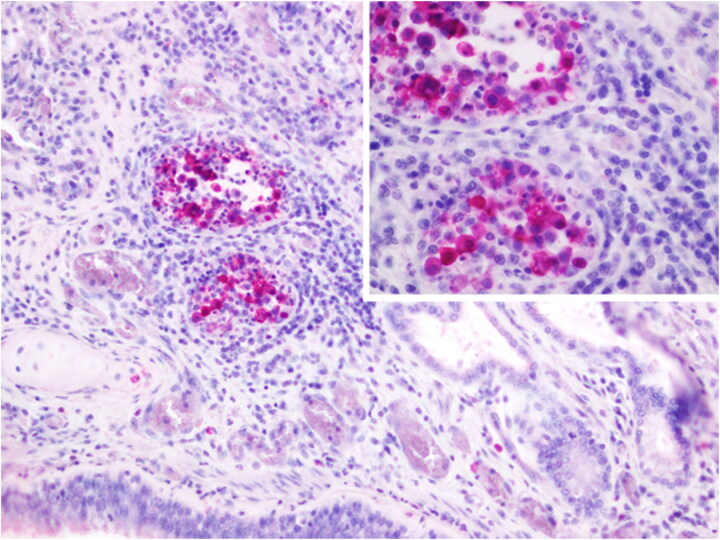
Lung. Male sea lion (245513). intranuclear and intracytoplasmic staining for Influenza A nucleoprotein in peribronchial inflammatory cells and cell debris in peri-bronchial glandular area. IHC against Influenza A nucleoprotein, 20×. Inset: magnification of the positive immunostaining. 60×.

The IHC revealed the presence of intralesional nucleoprotein antigen of AIV exclusively in the brain of both analyzed sea lions and in the lungs of the male (Figures 4C and D and 6). Specifically, positive immunostaining was observed in the nucleus, soma, and dendrite of neurons and the nucleus and cytoplasm of glial cells and other inflammatory cells (Figure 4C inset). Additionally, immunopositivity was also detected in a local area of peribronchial inflammation in the male sea lion. The brain from the male and the cerebellum from the female South American sea lions from the FFPE samples were positive for the avian influenza virus matrix gene of type A influenza virus and for the H5 hemagglutinin subtype. No detectable morbilliviral nucleic acid was detected in the analyzed samples. The overall information about necropsied animals is compiled in Supplementary Table S2.

## Discussion

In this study, we reported the mass and unusual mortality event in *Otaria flavescens* observed in Chile and its link with HPAI H5N1. The data indicate that the mortality started in January 2023, where in a month the stranded individuals overpassed the usual events in a calendar year before HPAI H5N1 detection. According to the NOAA, the current data reveals the occurrence of an unusual mortality event (UME), as evidenced by significant increases in morbidity, mortality, or strandings compared to historical records, along with temporal and spatial shifts in these patterns. Furthermore, the affected animals exhibit consistent or uncommon pathological findings, behavioral changes, clinical signs, or overall physical conditions, all of which align with the established criteria for identifying a UME (NOAA 2023).

To be linked with HPAIV events in avian should be interesting to compare the data observed in *Otaria flavescens* to other species of deceased animals commonly observed in that time period, such as pelicans, boobies, and gulls. Unfortunately, mass mortality events in wild birds were not registered as the data recorded for SA sea lions. This could be related to the difficulty of identifying the avian species and the size of sea lions simplifies its identification. The first mortality cases in avian were observed in early December 2022, where pelicans, gulls, and boobies, were quickly confirmed positive for HPAI H5N1. The first report of this virus in Chile was confirmed on December 05, 2022, from oropharyngeal and cloacal swabs of deceased pelicans (Ariyama et al. 2023). HPAIV H5N1 detection in *Otaria flavescens* seems to be under-detected, only 20% of the samples collected resulted positive for Influenza A virus. Up to date, around the world, the reports about marine mammal mortality caused by HPIAV H5N1 have been descriptive, and the success in the detection has been not included. The low percentage of positivity animals can be attributed to various factors, primarily associated with the sample collection, environmental conditions, and the pathogenesis of the virus. Swab samples may yield negative results due to delayed sampling, wherein the virus has potentially been eliminated from the targeted tissues, or because the virus could not reach the intended epithelial sites. Similar experiences with negative swabs and positive tissues have been observed in H5N8 infections detected in harbor seals on the German North Sea coast, in 2021(Postel et al. 2022). In addition, during the initial phase of stranding/mortality cases, a significant number of samples were collected from deceased animals without proper documentation of the date of death. Probably, some of these samples were collected several hours after the animals had died. Moreover, since the animals were found on the shore, there is a likelihood that the samples were exposed to seawater, and high temperatures, which could have affected the accuracy of the results. Despite this, reports indicate that seawater in this scenario could not affect the primary detection, the virus can persist for several days in seawater (Domanska-Blicharz et al. 2010; Hall et al. 2020). In the North of Chile, where most cases have been observed, the sea water’s average temperature during the study period is around 18–20 °C. Interestingly, studies conducted *in vitro* have shown that the H5N1 virus can remain viable for several days at these seawater temperatures (Domanska-Blicharz et al. 2010). As mentioned before, the deceased sea lions were also exposed to high temperatures on the beach. In the north of Chile, in coastal areas, the range of temperature is on average 18 °C − 25 °C most of the year, and in the Bio-Bio region between 10 °C − 20 °C. The temperature can also affect the survival of HPAIV or IAV in general and also have a role in the IAV transmission (Lowen et al. 2007, 2008). Even with high-temperature conditions, the inactivated virus should be detected by the molecular diagnostic (Shahid et al. 2009; Martin et al. 2018). Thus, the real-time RT-PCR results from swabs can be related to the pathogenesis in SA sea lions, as previously mentioned. In the necropsied sea lions, we also observed negative and positive to real-time RT-PCR in swabs.

However, despite the microscopic lesions observed in both necropsied South American sea lions, the antigen was exclusively immunolabeled in the lungs and brain of the male and the cerebellum of the female. This is compatible with the clinical manifestation for the male, but not evident for the female. The most conspicuous neurological syndrome was able to be observed in the male sea lion (Video 1).

These clinical signs were coincidental with a more severe and extensive histopathological lesions of the brain, such as severe multifocal to locally extensive areas non-suppurative encephalitis, and hemorrhages with necrosis of neurons and glial cells. In this scenario, the absence of detection in swabs, along with the presence of neurological evidence and the animal’s good body condition, is indicative of a possible acute form of the disease. In the female, despite there were not any neurological signs, there was a less severe and less extensive multifocal lymphoplasmacytic perivascular cuffing and hemorrhages in the cerebellum. The virus was detected in the female’s rectal and tracheal swabs, and her poor body condition suggests the possibility of a different syndrome affecting this animal or a more chronic phase of HPAIV H5N1 infection. There is extensive evidence of IAV viruses in pinnipeds before the HPAIV H5N1 panzootic, which include the subtypes (H7N7, H4N5, H4N6, H3N3, H1N1, H3N8, H10N7) (Runstadler and Puryear 2020). Most of the IAV cases reported in pinnipeds have been associated with clinical and pathological findings of pneumonia and primarily detected from the respiratory samples (Zohari et al. 2014; Bodewes et al. 2015.; Krog et al. 2015; Gulyaeva et al. 2018). In addition, in the majority of the cases, the source of the virus has been avian, except the H1N1pdm transmitted from humans (Goldstein et al. 2013; Runstadler and Puryear 2020). One of the most extended epidemics was documented in Europe in 2014–2015 caused by a H10N7 strain, which was limited to the respiratory system (Zohari et al. 2014; Bodewes et al. 2015; Krog et al. 2015). Interestingly, in 1980 an H7N7 strain was isolated in harbor seals (*Phoca vitulina*) which was also found in the brain. On the contrary, in concordance with our study, recent reports of HPAIV H5N1 in pinnipeds have been indicated with partial or extensive evidence of clinical neurological signs (Gamarra-Toledo et al. 2023; Leguia et al. 2023; Puryear et al. 2023). Here, we confirm based on molecular, pathological techniques the presence of HPAIV H5N1 in the brain and cerebellum of SA sea lions proving the neurologic tropism of the virus. The neurologic form of HPAIV has been extendedly reported in avian species but is not common in mammals (Klopfleisch et al. 2006; Foret-Lucas et al. 2023). Recently, HPAIV H5N1 was reported in several terrestrial wild carnivore species causing neurologic clinical signs finding de virus in the CNS in concordance with our results (Vreman et al. 2023).

Based on the overall results, we propose that most of the SA lions experienced an acute form of the disease, where the virus rapidly invaded the central nervous system (CNS), leading to neurological signs that caused stranding and rapid death, even more as a result of ataxia and paralysis of limbs many of them drowned and washed up dead on the shoreline. In some cases of per-acute infections, the excretion of the virus might not always be detectable, which could explain the lack of detection in certain instances. In this regard, it will be crucial to determine by serology the percentage of sea lions that have recovered.

One important question that needs to be addressed is whether transmission occurs from sea lion to sea lion or if the virus is only directly transmitted from avian sources. This inquiry, which was also raised by Gamarra Toledo et al. (preprint), in response to the identification of widespread stranding events in Peru, serves to challenge the previously established assumptions concerning direct avian transmission (Gamarra-Toledo et al. 2023). Recently, next-generation sequencing of several Chilean cases, including sea lions, other mammals, and avian species, suggests no direct transmission between sea lions, pointing towards transmission from avian sources (Pardo-Roa et al. 2023 preprint). Despite this, the epidemiological data suggest a potential transmission between sea lions, thus requiring further investigation.

Northern regions (Arica, Tarapaca, Antofagasta, Atacama, and Coquimbo) and the Biobio Region are the most affected areas. While this pattern could be partly influenced by the size of populations in those regions (Oliva et al. 2020), it does not fully explain the occurrence of cases in all regions.

After repeatedly observing wild sea birds interacting with SA sea lions in the field, we developed a hypothesis based on the shared food habitat as the most probable way of virus transmission between these two groups of species. Our hypothesis suggests that when positive HPAI-H5N1 wild avian species attack a school of fish to obtain prey, SA sea lions, also benefit from the abundance of food. This creates an opportunity for interspecies transmission. Interestingly, the regions with the highest number of positive cases are the only areas where both SA sea lions and wild birds share the same school of fish, particularly anchovies and sardines, which happen to be the preferred prey of abundant wild birds and SA sea lions in these regions (Neira and Arancibia 2004; Weichler et al. 2004; Ludynia et al. 2010). In contrast, in the other regions of Chile, SA sea lions primarily rely on a different diet consisting of larger prey such as hakes, salmons, and other demersal fish, which due to their sizes are unattractive to wild birds Oliva et al. 2020). As a result, the contact rates between sea lions and wild birds in these regions are relatively low. This pattern is evident in the mortality statistics of these regions, where no feeding habitat sharing is verified (Table 1). Another possible explanation for HPAIV H5N1 transmission could be the consumption of positive sick avian species. This hypothesis was previously proposed by Gamarra et al. in Peru after documenting an SA sea lion catching a seabird (Gamarra-Toledo et al. 2023). This mode of transmission has also been suggested in other mammals affected by HPAIV H5N1 (Krammer and Schultz-Cherry 2023), and thus cannot be fully discarded. However, it may not account for all the cases due to the high number of infections, and it is not a common behavior for SA sea lions to feed on carcasses of dead birds. In both scenarios, the high presence of HPAIV H5N1 in avian is needed which was effectively demonstrated in the figure spatial analysis (Figure 3). Additionally, it is important to mention that the most likely route of virus entry is through the digestive system, which requires further investigation as well.

The comprehensive analysis of epidemiological, spatial, pathological, and molecular data strongly supports the association between the mortality events and HPAIV H5N1, confirming the high susceptibility of SA sea lions to this virus. Despite this, the pathogen has proven challenging to detect in SA sea lions, particularly in swabs, possibly due to the neurological nature of the infection.

The most probable mode of transmission is from avian species during feed-sharing activities, a mechanism that appears to be highly effective. However, transmission between sea lions themselves is considered unlikely. Further research is imperative to fully comprehend the pathogenesis of HPAIV H5N1 in SA sea lions, including disease progression and tissue tropism, as well as the routes of transmission and potential sources of dissemination.

Other implications of this study to consider are the potential for sea lions to act as a source of the virus to humans, the importance of sea lion conservation, and the role of sea lions in transmitting the virus to avian species and potentially indirectly to poultry.

## Supplementary Material

Supplemental Material
